# 

**Published:** 1896-09

**Authors:** 


### CONTENTS—SEPTEMBER, 1896.—VOL. XII., NO. 3.

ORIGINAL CONTRIBUTIONS—
Puerperal Eclampsia. T. M. Yett, M. D. . . . . . . . . . . . . . 109
Child Crying in Utero. W. M. Powell . . . . . . . . . . . . . . 111
Tape Worm Diagnosis by Urinalysis . . . . . . . . . . . . . . 112
CORRESPONDENCE—
Letter from Dr. Q. C. Smith, including letter of Dr. Maclean . . . 113
Texas Health College Diplomas . . . . . . . . . . . . . . . . 116
A Protest . . . . . . . . . . . . . . . . . . . . . . . . . 116
SOCIETY NOTES—
Medico-Legal Society of Chicago, Dr. Lydston's paper. American Public Health Association. Meeting of American Association of Obstetricians and Gynecologists. Mississippi Valley Medical Association. Tri-State Medical Society. From Committee on Medical Societies. Tyler Medical Society . . . . . . . . . . . . . . . 117-133
Austin District Medical Society . . . . . . . . . . . . . . . 151
ABSTRACTS AND SELECTIONS—
Strangulated Hernia . . . . . . . . . . . . . . . . . . . . 135
EDITORIAL—
The Situation . . . . . . . . . . . . . . . . . . . . . . . 142
Death of Dr. Jerome Cochran . . . . . . . . . . . . . . . . 143
An Unmerited Slur . . . . . . . . . . . . . . . . . . . . . 144
The Nude in Medical Journalism . . . . . . . . . . . . . . . 145
A Practice Much Affected . . . . . . . . . . . . . . . . . . 146
The Screw Tightens . . . . . . . . . . . . . . . . . . . . . 147
Dr. William Henderson Wilkes . . . . . . . . . . . . . . . . 148
MEDICAL NEWS AND MISCELLANY—
Numerous Items . . . . . . . . . . . . . . . . . . . . . . . 151
THE JOURNAL'S EXCHANGE BUREAU . . . . . . . . . . . . . . . . 155
BOOK NOTICES . . . . . . . . . . . . . . . . . . . . . . . . 156
PUBLISHERS' NOTES . . . . . . . . . . . . . . . . . . . . . . 159

